# “It's OK for Me to Cry”: Client and Therapist Perspectives on Change Processes in SPEAKS Therapy for Anorexia Nervosa

**DOI:** 10.1002/jclp.23769

**Published:** 2025-01-13

**Authors:** Cat Papastavrou Brooks, Abigail Rennick, Randeep Singh Basra, Tony Lavender, Helen Startup, Anna Oldershaw

**Affiliations:** ^1^ SPIRED Clinic Sussex Partnership NHS Foundation Trust Worthing West Sussex UK; ^2^ Population Health Science, Bristol Medical School University of Bristol Bristol UK; ^3^ Kent and Medway All Age Eating Disorders Service North East London NHS Foundation Trust Maidstone Kent UK; ^4^ Health in Mind Sussex Partnership NHS Foundation Trust Worthing West Sussex UK; ^5^ Salomons Institute for Applied Psychology Canterbury Christ Church University Canterbury Kent UK; ^6^ Schema Therapy School and Brighton Psychology Service London UK

**Keywords:** Anorexia Nervosa, change processes, eating disorders, emotion focused therapy, psychotherapy, SPEAKS

## Abstract

**Introduction and Aims:**

Existing therapies for Anorexia Nervosa (AN) have limited effectiveness, necessitating the development of novel therapies and interventions. Hypothesizing and targeting clear mechanisms of change within treatment offer potential opportunities to improve them. The SPEAKS program aimed to develop, trial, and evaluate a therapy which targets key emotional and social factors known to be relevant in the development and maintenance of AN. The aim of the present study is to explore therapist and client experiences of change processes during the SPEAKS intervention, and what supported or inhibited these.

**Method:**

Semi‐structured interviews were conducted with sixteen female clients (in age range of 18–49) and six therapists; topic guides explored perceptions of client change processes. Thematic analysis was conducted on the data by two researchers.

**Results:**

Two themes and six sub‐themes were developed from the data. These were: “the impact on the eating disorder,” “change processes” (“emotional change” and “changing the self”), and “facilitators of and barriers to change processes” (“therapeutic relationship,” “clients’ emotional engagement,” “online delivery,” and “therapist lacking flexibility”). “Emotional change” involved an enhanced capacity for clients to tune‐in more, acknowledge, listen to, and express how they felt, and “Changing the self” represented a shift in how clients related to themselves, particularly the more vulnerable parts of themselves.

**Discussion:**

The findings of the present study provide support for the hypothesized mechanisms of change inherent within the SPEAKS therapy approach. This supports the robustness and validity of the intervention and lends support for further investigation of its effectiveness.

**Clinical Trial Registration:**

The study was registered according to the guidelines of the International Standard Randomized Controlled Trial Number Register (ISRCTN No. 11778891).

## 1 Introduction and Aims

### 1.1 Existing Treatments for Anorexia Nervosa

Anorexia Nervosa (AN) is characterized by disturbance of body image (or a persistent lack of recognition of seriousness of low bodyweight) and intense fear of gaining weight leading to self‐starvation and (in typical presentations) a significantly low body weight (APA 2013). It has one of the highest rates of mortality compared to other psychiatric disorders (Arcelus et al. 2011; van Hoeken and Hoek 2020) which is between five and six times as high as the general population (Arcelus et al. 2011; Crow et al. 2009; Murray et al. 2017). National Institute for Health and Care Excellence (NICE) recommended AN treatments for adults consist of either cognitive behavioral therapy (CBT), the Maudsley Anorexia Nervosa Treatment for Adults (MANTRA), or specialist supportive clinical management (NICE 2017), the latter of which was originally a control condition, found to be superior to CBT (Hay 2013). This is symptomatic of the inconclusive evidence base for psychological therapies targeting AN (Bulik et al. 2007), and lack of gold‐standard psychological therapies available (Murray et al. 2017); a recent network meta‐analysis of psychological interventions for adult outpatients with AN finding no “consistent first‐option” emerging from guidelines (Solmi et al. 2021).

Part of the issue might be a lack of understanding of the possible mechanisms of change in treatment (Wollburg et al. 2013), and therefore a failure to target known factors which maintain AN such as difficulties in emotion processing and regulation (Leppanen et al. 2022; Oldershaw et al. 2015; Puttevils et al. 2021; Wayda‐Zalewska et al. 2022; Wong et al. 2023). A systematic review of emotion‐focused treatments for AN found initial evidence supporting their acceptability and feasibility, but recommends further controlled studies to evaluate their efficacy, as well as more work examining mechanisms of action and treatment moderators (Sala, Heard, and Black 2016). Included studies with adult populations overwhelmingly evaluated either cognitive, cognitive based therapies or skills‐based therapies (e.g., cognitive remediation and emotional skills training, acceptance and commitment therapy) or behavioral therapies (dialectical behavior therapy, radically open dialectic behavior therapy, emotion acceptance behavior therapy) (Sala, Heard, and Black 2016). Only two studies evaluated emotion focused therapy (Sala, Heard, and Black 2016), a treatment aiming to support the processing of difficult emotions directly (without a cognitive or behavioral mediator), by attending to and increasing awareness of difficult emotions, making meaning of them by symbolizing them verbally, and transforming maladaptive emotions by activating adaptive ones (Ivanova and Watson 2014). One of these two studies, one was a case‐study of a single individual (Dolhanty and Greenberg 2009), and one evaluated emotion‐focused family therapy for children and adolescents (Robinson, Dolhanty, and Greenberg 2015); therefore are no evaluations of this therapy in treating AN in an adult population.

### 1.2 Targeting Change Processes in Treatment

Medical Research Council guidance on developing and evaluating complex interventions cautions that insufficient specificity in understanding the change process in complex interventions can mean that key variables are not successfully targeted in intervention development, leading to less effective treatments (Craig et al. 2011; Skivington et al. 2021). Change process research was developed to better understand how psychological therapy produces change for the client as part of developing more effective therapeutic interventions (Greenberg 1986, 1991). It can pay attention both to processes within the therapeutic space, and the temporal order in which they occur—crucially looking at both why and how change occurs in therapy (Elliott 2011). As such, it constitutes a bridge between outcomes research (assessing the impact of a course of therapy) and process research (which describes what occurs within the sessions), with most change process to date being quantitative and hypothesizing in nature (Elliott 2011). However, transdiagnostic and trans‐modal work around emotional processing highlights other change processes that are not well‐articulated or well‐targeted in other therapies. This means more qualitative work is needed to explore how therapies could facilitate emotion‐related aspects of the change process whether or not they are explicitly targeted in treatment (Elliott 2011). Qualitative work also presents a key opportunity to understand agreement and disagreement on the occurrence of change processes over a course of therapy from the perspectives of clients and clinicians, with client‐therapist agreement on the frequency of change indicators associated with positive therapeutic outcomes (Altimir et al. 2010). Qualitative work is particularly vital in understanding change processes as a path of the pathway to developing novel therapeutic treatments (Elliott 2011). This is because of its capacity to explore processes and experiences, remaining open to factors which have not been previously theorized by researchers. Once potential change processes have been explored in a more nuanced way for a particular therapy using qualitative methods, then quantitative work can be done (typically as part of an RCT) to understand whether these identified processes have a role to play in the therapy's effectiveness (Elliott 2010).

### 1.3 The SPEAKS Program of Research

The Specialist Psychotherapy with Emotion for Anorexia in Kent and Sussex (SPEAKS) research program aimed to develop, trial and evaluate an emotion‐focused psychological therapy intervention to treat AN in adults (Oldershaw et al. 2022). It utilized an interventionist causal model (Kendler and Campbell 2009), seeking to understand the mechanisms by which key factors could affect the development and maintenance of AN and be targeted in psychological treatment. Initial quantitative and qualitative work conducted during the SPEAKS intervention development phase (Phase One) focused on identified proposed objectives for change and how they might be targeted using “intervention mapping” (Oldershaw et al. 2022, 2024). Phase Two consisted of a single‐arm multi‐site feasibility study, with an embedded process evaluation with 46 participants consenting to trial participation, 42 entering the trial and 34 completing the 40 session course of SPEAKS therapy (Oldershaw et al. 2024). The study design meant that it was not possible to establish the effectiveness of the SPEAKS intervention (i.e., single‐arm feasibility), but 43% of clients who started treatment underweight were in remission at 12‐months (Oldershaw et al. 2024).

The present qualitative study forms one component of the SPEAKS program's range of process evaluation studies to understand how context, implementation, and change processes during the SPEAKS feasibility study contributed to the how the SPEAKS intervention was seen as having impact. It was conducted alongside a qualitative acceptability evaluation, which found SPEAKS to be an acceptable intervention for treating AN (Rennick et al. 2024), and a mixed‐methods evaluation of the process of emotional change within treatment analyzing therapy session recordings (Malik‐Smith et al. In prep).

Specifically, this paper focuses on exploring therapist and client experiences of therapeutic change processes during the SPEAKS intervention, and what supported or inhibited the facilitation of these.

## 2 Methods

### 2.1 Participants

**Figure 1:**
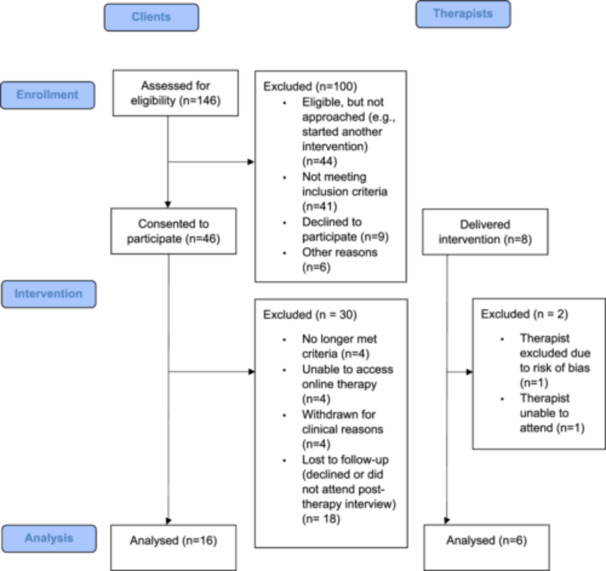
CONSORT.

Thirty‐four clients from two participating NHS trusts, North East London NHS Foundation Trust (NELFT) and Sussex Partnership NHS Foundation Trust (SPFT), were recruited to the SPEAKS trial. The SPEAKS therapy intervention was delivered by eight therapists across the two Trusts. Eligibility for inclusion in the present study was to have either completed or delivered SPEAKS therapy, giving a total of 34 eligible clients and seven therapists. Client and Therapist eligibility criteria for the SPEAKS study is described in Table 1. Clients were allocated to a particular trial therapist based on therapist availability, and were not able to choose their therapist. Sixteen clients were interviewed, (four from SPFT, twelve from NELFT) as well as six therapists from both trusts (see Figure 1). Participants were not asked why they declined an interview.

**Table 1:** Client and therapist eligibility and exclusion criteria.

	Eligibility criteria	Exclusion criteria
*Clients*		
	Are referred into Kent or Sussex EDS and meet service criteria (e.g., registered with a local general practitioner). Meet Diagnostic and Statistical Manual 5 Criteria for Anorexia Nervosa or OSFED (Other Specified Feeding or Eating Disorder) of Anorexic type. Are aged 18 or above. Have body mass index (BMI) > 15 kg/m^2^. Have sufficient English language abilities to complete a talking therapy.	Rated as “High Risk,” or as “High Concern” in weight criteria, on the MARSIPAN Guidelines for adults with eating disorders (i.e., BMI < 15 kg/m^2^; weight loss > 500 g for two consecutive weeks). Considerable psychological risk, including active suicidal thoughts and plans. Comorbidity requiring treatment priority. Alcohol/substance use disorder. Participating in another treatment trial. Diagnosed Intellectual disability impeding ability to use therapy. Pregnant
*Therapists*		
	Are a specialist eating disorder therapist > 3 years experience. Work in Kent All Age EDS or Sussex EDS. Have specialist training in an experiential dialogical self chairwork model (eg, emotion‐focused therapy (EFT), schema therapy (ST), compassion‐focused therapy).	

Client and Therapist demographics for this qualitative study are presented in Tables 2 and 3 respectively. All client participants were female, and 14 (87.5%) were White‐British. For client demographics for the feasibility study, please see the trial paper (Oldershaw et al. 2024).

**Table 2:** Client demographics.

Characteristic		*n*
Gender	Female	16
	Male	0
		
Age	18–19	1
	20–29	7
	30–39	4
	40–49	4
		
Ethnicity	White ‐ British	14
	Mixed ‐ White & Black Caribbean	1
	Not stated	1
		
Illness duration (years)	0–3	4
	3–6	3
	6–10	1
	10–19	5
	20–29	3
		
Employed	Paid/self‐employed	11
	Unemployed	1
	Student	1
	Volunteer	1
	Homemaker	1
	Unknown	1
		
Previous psychological treatment?	Yes	13
	No	3
Any treatment at all (including pharmacological)	Yes	16
	No	0

**Table 3:** Therapist demographics.

Characteristic		*n*
Gender	Female	5
	Male	1
		
Professional Training	CBT Therapist	1
	Clinical Psychologist	4
	Counseling Psychologist	1

### 2.2 SPEAKS Intervention

SPEAKS is a psychotherapy for adults with AN, aimed at targeting emotional experience as a core hypothesized maintaining factor (Oldershaw et al. 2015, 2022; Oldershaw and Startup 2020). It is an integrative therapy drawing on experiential, dialogical‐self and chairwork models, predominantly Schema Therapy (ST) and Emotion Focused Therapy (EFT) and is described in greater detail elsewhere (Oldershaw et al. 2022, 2024; Oldershaw and Startup 2020, 2023). It is a weekly outpatient 1:1 intervention lasting 9–12 months, with up to two follow‐up sessions. Although the therapy had been designed for in person delivery, as was the case with most therapeutic interventions at this time, the abrupt onset of lockdown meant that the team had to adapt and deliver the treatment online. There were no specific adjustments made to the content of the therapy, but the delivery was via video platform.

### 2.3 Procedure

All participants completing SPEAKS and therapists delivering SPEAKS therapy were invited to take part in post‐therapy qualitative interviews once their involvement with the trial was complete. One therapist (AO) was not invited to take part due to her dual role of chief investigator and intervention developer. Semi‐structured interviews were conducted via video call by the research assistant on the SPEAKS feasibility trial (RSB). Interviews lasted up to 1 h, and were transcribed verbatim, although anonymized to maintain confidentiality.

Interviews explored client and therapist experiences of the therapy, including how acceptable they felt the intervention had been, facilitators of and barriers to intervention delivery, and which aspects of the intervention had been most helpful in effecting change. Topic guides were developed by authors AO and RSB and included an adaption of Elliott and Rogers's (2008) Client Change interview schedule (Elliott and Rodgers 2008) to explore change processes, alongside questions about the acceptability to the intervention (see Appendices 1 and 2). Analysis of acceptability data were carried out in a counterpart paper to this one (Rennick et al. 2024).

Ethical approval was granted by London‐Bromley Research Ethics Committee (Ref.: 19/LO/1530). Written informed consent for participating in the SPEAKS study (including qualitative interviews) was sought for both therapist and client participants before enrollment in the study and participation in qualitative interviews was verbally confirmed at the end of therapy. The study was registered according to the guidelines of the International Standard Randomized Controlled Trial Number Register (ISRCTN No. 11778891).

### 2.4 Analysis

A reflexive thematic analysis methodology was used to interpret the data (Braun et al. 2019; Braun and Clarke 2006). Because a single set of interviews were conducted as part of SPEAKS which covered both questions about the acceptability of the intervention (Sekhon, Cartwright, and Francis 2017), and the client change interview schedule (Elliott and Rodgers 2008), interview data pertained both to acceptability and change processes. It was therefore decided to code this data set as a whole (and separate later in analysis), as opposed to splitting each interview into two‐parts at the outset, since participants spoke about change processes as a response to questions on acceptability and vice‐versa.

One researcher (AR) read all transcripts, and another researcher (CPB) read 30% of transcripts to familiarize themselves with the data. Similarly, during coding, AR coded all transcripts, and CPB coded 30% of the transcripts. Both researchers inductively coded the transcripts, that is codes were developed from the data, not determined in advance. Both researchers (AR and CPB) met regularly to review and discuss codes, exploring any differences between their coding. This was aimed at enhancing their reflexive understanding of the meaning and application of the codes—as opposed to reliability—in line with the epistemological standpoint of reflective thematic analysis (Braun et al. 2019).

Following coding, 45 codes (and the data which related to each code) were subsequently grouped into nine initial domain summaries (Braun et al. 2019). These were descriptive and covered a small part of either clients or therapists’ experiences of the intervention (e.g., therapist experience of supervision). At this point, analysis was split—where a domain summary most related to the acceptability of SPEAKS components and processes it was written up as part of our acceptability analysis, led by AR (Rennick et al. 2024), and where it mostly related to change processes it was included as part of this paper. A coding tree for both papers is included as Appendix 3.

The two final reflective themes of this paper were generated by both researchers reflecting on and discussing the causal processes by which SPEAKS was experienced as beneficial to clients, synthesizing domain summaries into a single model able to describe with a sufficient level of granularity how individual SPEAKS components brought about change for clients. Although initial coding was inductive, development of themes involved researchers discussing and integrating existing conceptual frameworks, such as those relating to process evaluation components (Skivington et al. 2021); impact on outcomes, change processes and facilitators of and barriers to change processes. This combination of initial inductive coding, but deductive synthesis of themes is recommended in cases where the research question means that findings can best be illuminated and understood in the context of a pre‐existing theoretical framework (Braun and Clarke 2006; Varpio et al. 2020). Themes and sub‐themes were reviewed, discussed, and amended by both researchers. Minority perspectives (i.e., where one or more participant disagreed with the majority) were included in the paper, to ensure that potential harms or disadvantages of the SPEAKS therapy which applied only to a small minority could be understood.

### 2.5 Reflexivity

Two researchers were involved in analysis to offer a broader range of perspectives on this data and enable dialog and critical discussion between researchers to enhance reflection and interpretation, in line with the epistemological standpoint of reflexive thematic analysis (Braun et al. 2019).

Neither researcher conducting analysis for this paper (CPB and AR) was involved in intervention development, delivery, or data collection, and did not familiarize themselves with the SPEAKS intervention protocol or associated publications before analysis. By contrast, the SPEAKS study research assistant (RSB) conducted the interviews and knew all the participants throughout the study, as well as the intervention content. The chief investigator (AO) had developed the intervention. Emerging themes were not shared with RSB or AO until after analysis was completed, to avoid their perspective (as researchers familiar with the proposed change processes) being unintentionally privileged in analysis. All researchers except CPB had clinical roles alongside conducting research, which would have led to greater insight into practical and theoretical aspects of SPEAKS therapy.

Both CPB and AR are young white women and given the range of ages in the study in particular—might have been less sensitive to issues around the differential impact of the intervention on people of different ages, genders, or races. During analysis meetings, both researchers spent time discussing and critically examining their own positionality in relation to that of participants and how this could have limited their interpretations of the data. However, the similarities between the researchers also could have caused them to feel more comfortable sharing personal information that informed their analysis (such as first‐hand experiences of mental health issues alongside discrimination as a result of age or gender) enabling more vulnerable and therefore richer and more reflexive analysis meetings.

## 3 Results

Two main themes and six sub‐themes were developed from the data.

**Figure 2:**
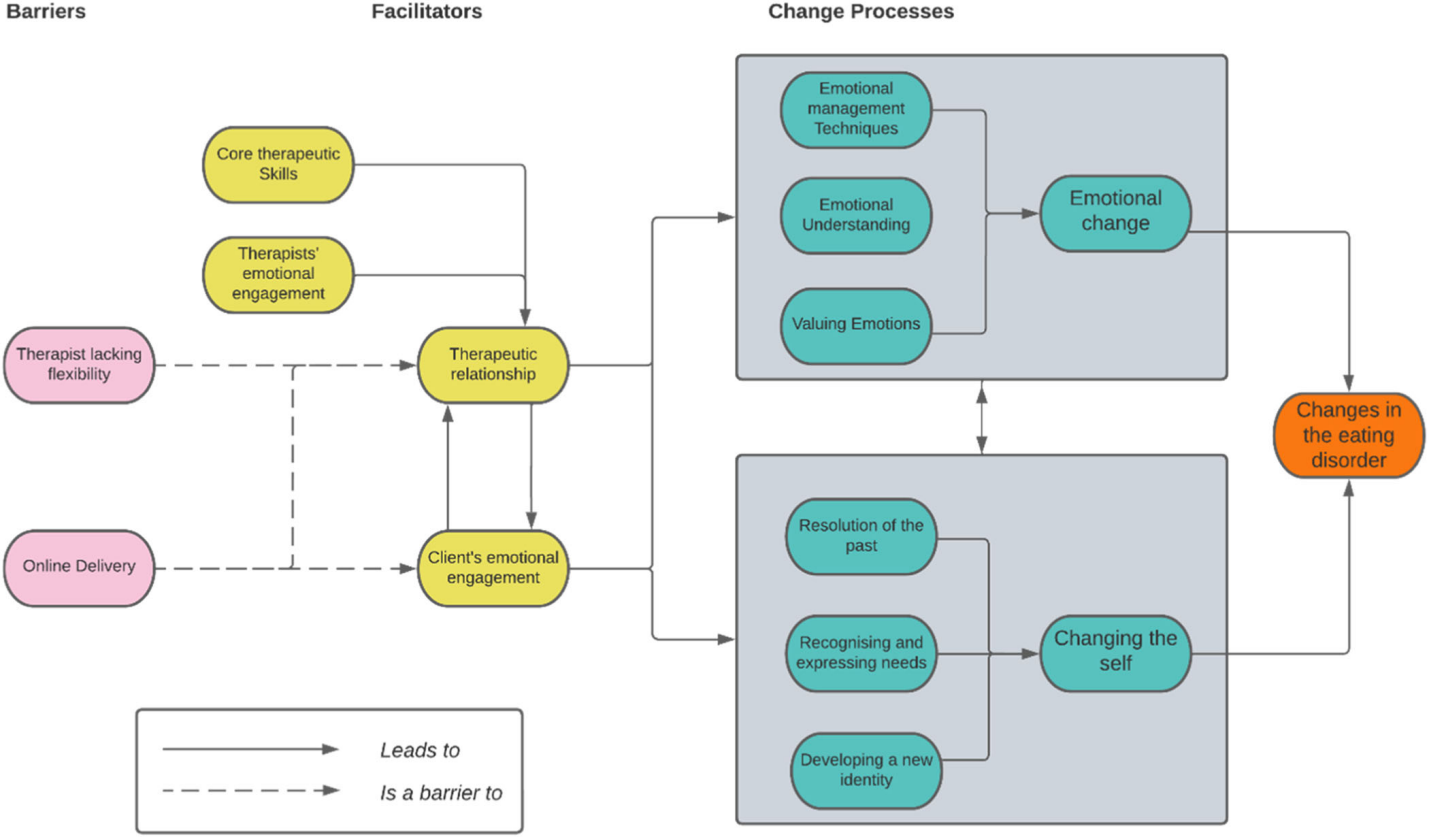
SPEAKS change processes.

Taken together, themes constitute a model of how the SPEAKS therapeutic intervention facilitated change in client's eating disorder symptomatology, through two core change processes: emotional change and changing the self. Facilitators of and barriers to this change were also considered and are described (See Figure 2). The lines in this model do not represent temporal or even necessarily causal relationships (which are beyond the scope of this paper to evaluate), but rather the factors whose presence was seen as necessary for other processes to occur (and often constitute the context within which those changes took place, as opposed to the causal instigator of those changes).

### 3.1 Experiences of Change and Change Processes

The impact of SPEAKS on food, eating and physical health reported varied and in part reflects the heterogenous nature of ED symptoms. Some clients felt that they had conquered their relationship with food, eating, and their bodies and were in remission from AN evidenced by removal of rules around food and exercise, not using Fitbits, being able to try new foods and foods they were particularly scared of (“fear foods”), getting physically healthier, and noticing a clear difference in their appearance. Another proportion of both therapists and clients felt that the ED was not entirely conquered and the impact of SPEAKS was more of a “work in progress.” Only one client felt pessimistic about recovery due to their belief that issues around food and body image were too heavily ingrained to change.

[I]t's really benefited me massively. I'm in remission from anorexia now. So, that was kind of the result that was hoped for, and it's worked. It's been good.

– Client, Female, 31

These changes in the eating disorder were seen to be caused by two central change processes experienced by participants: “Emotional change,” and “Changing the self.”

#### 3.1.1 Emotional Change

Client participants reported that before receiving SPEAKS they had attempted to hide or suppress emotions, putting them in a box which ran the risk of “overflowing.” After SPEAKS they were able to tune‐in more, acknowledge, listen to, and express how they felt. This emotional change was attributed to a range of different mechanisms.

Firstly, both therapist and client participants valued the techniques for emotional management gained through SPEAKS, such as challenging feelings, practising self‐care, sitting with painful emotions, whilst avoiding unhelpful coping mechanisms.

I think it's made me be a bit more confident in sitting with negative emotions and just, “OK, you're not feeling very good at the moment, what's actually going on here? You're feeling anxious.

– Client, Female, 47

They also discussed the importance of increased emotional understanding brought about by the therapy, extending not just to their own, but to others’ emotions. This process was mirrored by therapists’ accounts that SPEAKS had given them better understanding of both themselves and of clients.

One thing I want to add actually, it's not only helped me understand clients, but it's helped me understand myself better So a lot of what we use, you can easily apply to yourself, so I've become much more aware of the different parts of myself and who is at play and what I need.

– Therapist, T006

Changing the valuation of emotion was the perceived core mechanism by which SPEAKS changed clients’ relationship with their emotions. Clients described realizing that feeling upset, low, resentful, or angry was not necessarily negative, and could be reasonable ways to respond to a situation, guiding useful action. This in turn increased their willingness to be emotionally vulnerable with others. Both clients and therapists attributed this change to validation and legitimizing clients’ emotions and emotional responses within SPEAKS therapy.

I think I thought to show your emotions was a sign of weakness. Whereas now my view is more, actually, no, sometimes it can be sign of strength to show your vulnerabilities and it takes guts to show your vulnerabilities. So that's a real shift for me.

– Client, Female, 40

I feel like I'm able to express myself more, not just obviously to myself but you know, to other people that it's OK for me to feel this way and I, you know, it's OK for me to cry and to get my feelings out basically.

– Client, Female, 36

#### 3.1.2 Changing the Self

The other SPEAKS change mechanism described was in how clients understood and related to themselves. This was linked to emotional change, in that clients were better able to understand and relate to the more vulnerable or emotional part of themselves.

Clients and therapists overwhelmingly expressed beliefs that SPEAKS was able to get to the heart of issues and vulnerabilities and that this facilitated understanding of the self. Some clients reported initial reluctance to discuss their childhood in therapy yet found understanding the contribution of past events to their issues enabled identification of underlying behaviors, triggers and core beliefs pertaining to their ED difficulties. Clients described how insight into these factors generated a greater sense of control in their current lives, and reduced self‐criticism and guilt as they acknowledged the lack of control they'd had over past events.

But I really, really do believe with SPEAKS and it really shifting my perspective in terms of getting to the heart of my vulnerability and connecting to my childhood self and where that behavior all came from and understanding the boundaries I need to put in place for certain people and all that sort of stuff, it's really helped shift things.

– Client, Female, 29

Both therapist and client participants felt that SPEAKS enabled identification, expression, and action in accordance with needs, contrasting previous anxieties that this would either harm to others or result in rejection.

Most clients reported increased self‐confidence, less self‐doubt, increased inner strength, feeling “way more comfortable in [their] own skin”, and reduction in experiences of shame and guilt as a result of understanding their past better. Therapists also noted a reduction in the critical relationship towards the self in their clients, their growth in confidence and increased self‐compassion, leading to behavioral changes.

I think maybe I'm more confident and, yes, I guess more self‐assured…I think it's probably because I feel more deserving, and worthy of the external praise and kind of validation.

– Client, Female, 21

Both participant groups understood reduced self‐criticism and increased self‐compassion as connected to a growing sense of self or identity that was no longer about an ED. Clients appreciated how using a separate chair to embody the ED part helped them separate from the ED as an individual and rediscover their identity. Clients and therapists reflected on the value of using chairs to talk to parts of self previously excluded.

Instantly, after we had done the chair work, I felt like a weight lifted…I was able to get things out that I was holding in just by kind of like taking myself out of it. Making its own identity instead of it being part of me.

– Client, Female, 31

What I would say about the chair technique is that it allows the person to no longer closet or side‐line different parts of who they are, but to really understand themselves. And I think that can never be a bad thing.

– Therapist, T005

### 3.2 Facilitators of and Barriers to Change Processes

#### 3.2.1 Therapeutic Relationship

The most significant facilitator of SPEAKS change processes (experienced by both clients and therapists) was a strong therapeutic relationship. Clients repeatedly spoke about the creation of a safe space as necessary for SPEAKS therapy to work, potentially elevated because of the inherently challenging emotional work. Therapist/relationship characteristics such as kindness, compassion, empathy, and trust were particularly highlighted.

It's all about the relationship with your therapist, isn't it? And I just found I got on really well with her and I had that trust. I don't think I could have done it with somebody that I didn't have 100% trust in.

– Client, Female, 40

SPEAKS therapists had to learn a new therapeutic model different from previous experience in providing other NICE concordant ED therapies. The learning process of SPEAKS therapy tended to be conceptualized by therapists as “stacking” an additional layer on top of core therapeutic skills. Compared to working within other frameworks such as CBT, the centering of these core skills was key to how therapists felt they facilitated change processes for clients.

I think within the SPEAKS model it's easier as a therapist to hold that kind of position for truly listening than it is in some of the other evidence‐based models where there's a bit more pressure to be getting on with some tasks. There's something about SPEAKS that allowed, I feel allowed me to at times just settle back into basic therapy skills or counseling skills, however you would want to call it.

– Therapist, T003

The importance within SPEAKS of developing and sustaining a strong empathic relationship with clients, that focused on “being with” emotion, required additional emotional resources from therapists beyond usual practice. As such, therapists described SPEAKS as being more demanding emotionally than other therapies.

[t]he sessions are more emotionally demanding, they just are. You're following their emotion. Your own emotions do that. I couldn't quite imagine what it would be like to have a full caseload of SPEAKS clients, I think I, you know, that might be quite emotionally demanding.

– Therapist, T001

#### 3.2.2 Clients' Emotional Engagement

As discussed, engagement with emotion to recognize, experience and shift in the valuing of emotion was core to therapeutic change. However, where emotional engagement was partially or fully blocked, this constituted a significant barrier to the process, leading to increased feelings of vulnerability after sessions. This was reported to be particularly relevant for “difficult” emotions, which in some cases, had not been faced in depth in a long time.

There were times when I thought, “Oh, do I really want to open this Pandora's box?” Do you know what I mean? “Do I really want to go there, because it's making me feel so unhappy?”

– Client, Female, 40

Despite this, many were confident that this difficult emotional process supported their progress in challenging the ED.

Probably the only way to describe it is a little bit like an emotional rollercoaster. Some weeks would be absolutely fine. And then other weeks it would feel like you'd been hit with a brick. But you definitely felt like you were doing, again you felt like you were doing really hard work which made it feel like it was worth it

– Client, Female, 27

#### 3.2.3 Online Delivery Difficulties

SPEAKS was initially developed for face‐to‐face delivery but was forced online due to the Covid‐19 pandemic. There was a majority opinion across clients and therapists that online delivery was difficult and could be a potential barrier to change, particularly if clients did not have access to a laptop or high‐quality internet. Some therapists felt online delivery potentially hampered the how well the practical SPEAKS techniques worked, such as chair work and the use of toys/objects. They felt more experiential sessions would have benefited from being face‐to‐face.

Online delivery was also seen as a barrier to client‐related factors necessary for change processes, impeding vulnerability in the therapeutic space, and emotional engagement with treatment due to worries about the privacy of the space.

I think there's an element of me that has at times, not always, struggled to get to that vulnerable place because I'm kind of half, even subconsciously, aware that somebody is there that could be listening.

– Client, Female, 29

#### 3.2.4 Therapist Lacking Flexibility

SPEAKS (in line with other complex interventions) is designed for flexible delivery to meet client needs. As such, only techniques considered a good fit are utilized. Fidelity to this was compromised where a minority of therapists were overly didactic and did not adjust techniques to target change for each client, instead insisting that the client needed to engage with techniques (such as using toys to represent parts of the self) that they stated had felt patronizing and unhelpful for them. This negatively impacted the potential impact of the therapy. Clients reported damage to the therapeutic relationship (a key facilitator of SPEAKS change processes) as they did not feel their opinions were heard by their therapist (e.g., regarding some SPEAKS techniques not helping them, requests for adjustments to session frequency, or reduced BMI focus), all in clear contrast to the SPEAKS model.

I was kind of saying I didn't want to do that. But it was like no, well it's part of the study so we're doing it

– Client, Female, 21

## 4 Discussion

This study investigated how therapists and clients perceived the psychotherapeutic change processes associated with a novel emotion‐focused psychotherapy for adults with AN.

Most clients interviewed reported a positive impact of SPEAKS therapy on the ED; reporting the illness was in remission, or that they had made significant changes. This finding can be contextualized by quantitative analysis of clinical outcomes of the SPEAKS therapy, with significant decreases found in ED symptomatology of very large effect (Oldershaw et al. 2024). Despite the SPEAKS trial small sample size and single‐group design, this is promising, especially within the context of length of illness duration of the sample (9.0 years on average) and given a recent network meta‐analysis demonstrating that current NICE recommended treatments for AN do not outperform comparators, including treatment as usuanl (Solmi et al. 2021).

### 4.1 Change Processes

SPEAKS was developed to specifically target emotional processes (Oldershaw et al. 2022), thus the majority of participants identifying changes in client relationships with emotions is a valuable finding. Most participants felt this change had occurred principally due to an increased valuation of difficult emotion, seen as both intrinsically helpful—but also enabling increased vulnerability with others. It is recognized that emotional self‐regulation abilities are negatively impacted by environments where there are negative perceptions of vulnerability and emotional openness (Borelli et al. 2019), and that beliefs about emotion are linked to emotion regulation, well‐being, and psychological distress (De Castella et al. 2013, 2018). Improved beliefs about emotions have been previously associated with recovery from AN (Oldershaw et al. 2012).

Valuing emotion as a signaller for unmet needs is a feature of EFT, enabling improved understanding and expression of emotion leading to opportunities to meet previously hidden needs (Pos and Greenberg 2007). This is derived from a functional, evolutionary understanding of emotions as a source of important information about ourselves, others and our environments (Keltner and Gross 1999). For SPEAKS participants, valuing emotions (including those more uncomfortable to be with) was connected with increased understanding of, and compassion towards, the self, which may also reflect the reported shift in shame, considered central in the development and maintenance of AN (Blythin et al. 2020). It aligns with the SPEAKS proposition that building the “healthy adult” which can offer self‐compassion and acceptance is a core change process (Oldershaw and Startup 2020). It suggests the successful integration of EFT and ST techniques within SPEAKS and reinforces the value of such psychotherapeutic theory and practice.

Of note is that these findings are in line with the a priori SPEAKS model of change, hypotheses and goals, which guided the development of the intervention and are described fully in the SPEAKS protocol paper (Oldershaw et al. 2022). They mirror qualitative SPEAKS intervention development work which highlighted the need to “see emotions differently” and connect with emotions and self as core processes in recovery and growth (Drinkwater et al. 2022) and which shaped the SPEAKS intervention. It also reflects other work describing the discovery of the “real self” as central to recovery (Williams, King, and Fox 2016). Participant descriptions suggest successful facilitation of such change processes within the clinical application of SPEAKS, bridging the difficult to achieve gap between the empirical and clinical (Kazdin, Fitzsimmons‐Craft, and Wilfley 2017).

### 4.2 Facilitators of and Barriers to Change Processes

The therapeutic relationship was seen as key to facilitating clients’ engagement with SPEAKS. Therapeutic alliance has been frequently found to be a mediator of therapeutic change in systematic reviews, both in psychotherapy generally (Baier, Kline, and Feeny 2020), and in eating disorder treatment (Zaitsoff et al. 2015), at least partly because it contributes to treatment retention in a population with high drop‐out rates (Bandini et al. 2006; Clinton 2010; Morlino et al. 2007; Zaitsoff et al. 2015). Although therapeutic alliance is a mediator of change for many different psychotherapies (although notably not for CBT and eating disorders) (Brown, Mountford, and Waller 2013, 2014), which is one of only three NICE recommended interventions (NICE 2017), SPEAKS use of the therapeutic relationship is more significant than just protecting against drop‐out. As we have described in our model, within SPEAKS having a good therapeutic relationship creates the environment in which the other change processes can occur.

It is of note that within the SPEAKS therapy, there were no client initiated therapeutic drop‐outs (Oldershaw et al. 2024). Therapist participants felt that SPEAKS required more of their “core therapeutic skills” such as empathy and active listening to enable rapport building than they used in other therapies. It might be that in elevating the therapeutic relationship as a “core task” (Oldershaw and Startup 2023), SPEAKS has been able to tap into a key mechanism of change within EFT (Nødtvedt et al. 2019) not adequately utilized in other approaches (Crits‐Christoph et al. 1991). The facilitation of such processes further suggests EFT and ST were useful approaches to adopt and integrate in the development of SPEAKS given that the therapeutic relationship is facilitated by empathic attunement, emotional responsiveness and therapeutic presence, concepts derived from EFT (Elliott and Greenberg 2007; Stiegler, Molde, and Schanche 2018; Watson 2018), and the ST stance of “limited reparenting” (Flanagan, Atkinson, and Young 2020; Gülüm and Soygüt 2022).

On the flip side, progress was perceived as impeded when difficulties with emotional engagement existed. Individuals also identified how tough it can be to focus on some emotional material at certain points. This highlights the importance of motivation and timing of emotional processing work—especially where, for example, individuals may have endured trauma—within a therapeutic process and that having some degree of readiness, openness and willingness to engage in emotion‐based work might be a pre‐requisite for engagement in SPEAKS for both clients and therapists. It also highlights the need for an individualized approach, sensitive to client needs at each point in therapy, with session foci collaboratively agreed as is proposed in SPEAKS (Oldershaw and Startup 2023).

The need for such an approach is further supported by the negative experiences reported herein from clients where therapy was too didactic or inflexible. The application of specific techniques too rigidly was reported to undermine the therapeutic relationship and consequently outcomes. SPEAKS's guidebook approach specifically recognizes the fact that a too rigid application of therapy or fidelity makes psychotherapy less useable and effective (Cook, Schwartz, and Kaslow 2017). Unwillingness to adapt therapy according to client preference constitutes a deviation from the intended delivery of SPEAKS and is reflected in adherence data for a minority of participants (Oldershaw et al. 2024). This issue could be mitigated through improvements to therapist training, including awareness of the flexibility of intervention delivery, in contrast to more manualized therapies.

Therapists described finding it more emotionally draining to deliver SPEAKS than other therapies, because of the level of emotional engagement required. Although clinician burnout includes emotional exhaustion as a key component (Morse et al. 2012), studies have also found that emotional connection, and close emotional relationship with clients can mitigate the effects of burnout within ED (Graham et al. 2020) and other health services (Cain et al. 2017). Despite requiring more emotional resource to deliver than other therapies, SPEAKS could alleviate, not exacerbate, staff burnout. In addition, therapists would not be expected to have a full caseload of SPEAKS clients, who of note, are also some of the more chronically unwell clients presenting to ED services, thus associated with additional stressors. This is supported by finding that therapists in general found SPEAKS acceptable and felt well supported by the supervision they received as part of SPEAKS (Rennick et al. 2024).

### 4.3 Strengths and Limitations

Eligibility for this study required completion of SPEAKS therapy. Thirty‐four clients completed therapy, however only 16 were interviewed. The quantitative follow‐up for SPEAKS was 88% across all follow‐up appointments and both sites (Oldershaw et al. 2024), therefore there appears to have been a reluctance from some participants to engage with qualitative data collection specifically. This was perhaps due to the length of involvement in the trial and the additional burden of this extra follow‐up appointment; however, reasons such as dissatisfaction with the therapy may have led some participants to choose not to complete interviews. Participants who did not complete the therapy were not invited to interview. These factors combined presents a potential missed opportunity to gather the voices of clients who might have been critical of SPEAKS. The homogeneity of the sample (14 out of 16 clients were White‐British, and all were female) presents an additional barrier to the generalizability of the study findings. However, the sample size of this study is comparable to other qualitative research studies conducted as part of a feasibility trial of a novel intervention for eating disorders (Griffin et al. 2023; Keeler et al. 2022; McDonald et al. 2021).

Utilizing the same qualitative data set to fulfill two differing research aspects of a process evaluation (to understand change processes, and to assess the acceptability of the intervention) but conducting the analyses in tandem, is a key strength of the approach we took (Skivington et al. 2021). By developing topic guides which incorporated both acceptability and the client change interview schedule (Elliott and Rodgers 2008), and analyzing these together we were able to draw connections between the aims of the two papers; for example between how acceptable clients found the emotional demands of engaging with SPEAKS (Rennick et al. 2024), and the emotional process by which SPEAKS was hypothesized to work, as well as increasing the coverage of our coding process.

A variety of researchers collaborated on this paper—with different relationships both with SPEAKS participants and the intervention itself. The authorship team contained both clinical and nonclinical staff, including SPEAKS intervention developers (AO, HS and TL), knowledge of participants and the intervention through data collection (RSB), and no familiarity with either the intervention or participants (CPB and AR). This approach sought to reduce the risk of researchers aligning their interpretations of data on their understanding of the hypothesized change mechanisms, which the researchers might have taken as authoritative and thus impaired their capacity to develop new interpretations of the data. However it may have led to some things being missed in the analysis. The combination of researchers conducting analysis with minimal prior knowledge, but in frequent dialog with researchers with different varieties of knowledge, was hoped to mitigate this issue by ensuring that multiple interpretations of the same data were considered and offered and broaden the range of possible perspectives on the data (Braun et al. 2019). The topic guides for interviews were developed based on the Client Change Interview utilizing specific questions and themes that are advised to focus on, such as Valued Psychotherapeutic Targets and Techniques. Questions began open ended to give clients an opportunity to talk about change in a broad sense; indeed behavioral change around eating or in relationships for example were discussed for example, the first paragraph of “3.1. Experiences of Change and Change Processes.” The interviews did go on to ask about features specific to the SPEAKS intervention such as emotions and key therapeutic techniques to assess whether these added anything to the change process in particular, as these were psychotherapeutic targets and techniques within the intervention. Participants were not directly asked about cognitive or behavioral change although these were often introduced by clients within the interview format. Whilst we feel we took a valid approach, we recognize that we may of course have missed some of the potential change processes and thus this is a potential limitation in the breadth of the conclusions that can be drawn.

Both CPB and AR are young white women, and especially given the difficulties that men (Thapliyal, Mitchison, and Hay 2017; Wooldridge and Lytle 2012) and racially minoritized groups (Bodell et al. 2018; Gordon et al. 2006) face accessing appropriate treatment, blind spots on their part, might have led to the neglect of relevant factors. However, given the homogeneity of the sample for this qualitative study, it seems more likely that issues around lack of inclusion of the perspectives of people of color occurred at the stage of recruitment into the study, as opposed to analysis.

Because of the single‐arm study design and the small sample size of client participants in this qualitative study (*n* = 16), we did not think it would be meaningful to separate the data during analysis by factors such as age, duration of AN, previous experiences of therapy, or co‐morbidities, to draw conclusions about the impact of SPEAKS for different groups. However we acknowledge that the experience of SPEAKS would likely have been shaped by these and other complex factors, which would be important to explore in trials of this therapy.

No attempt has been made in this study to link participants’ qualitative data to the outcome measures or other data collected, such as changes in emotional state (Malik‐Smith et al. In prep; Pascual‐Leone 2018). Given that this analysis aimed to understand the mechanisms by which the intervention may have worked, and the contextual factors affecting this, differences between those who had benefited from the intervention or not in terms of how they appraised the process of change in the intervention, was not considered a major aim. This could be the subject for future analysis using existing data, of potentially significant value given that (at *n* = 22) this represents a medium‐sized qualitative data set for a novel psychological treatment, or potentially could be explored qualitatively in an RCT of the SPEAKS therapy.

## 5 Conclusion

This study found both clients and therapists felt improvements to eating disorder symptoms and behaviors brought about by SPEAKS therapy were linked to two perceived change processes; emotional change and changing the self. Emotional change was a process of clients increasing their ability to experience, recognize and value emotion, leading to recognition of one's needs, and better understanding of and connection to the self. Facilitators of this process included the therapeutic relationship, whilst barriers were didactic therapeutic approaches and online delivery. The emphasis on emotional change processes found in the data aligns with hypothesized mechanisms of change in the SPEAKS model and how changes in eating disorder outcomes can be facilitated in therapy. The findings of the present study provide support for the hypothesized mechanisms of change inherent within the SPEAKS therapy approach. This supports the robustness and validity of the intervention and lends support for further investigation of its effectiveness.

## Ethics Statement

Ethical approval was granted by London‐Bromley Research Ethics Committee (NHS Rec Reference: 19/LO/1530). Written informed consent for participating in the SPEAKS study (including qualitative interviews) was sought for both therapist and client participants before enrollment in the study and participation in qualitative interviews was verbally confirmed at the end of therapy.

## Supporting information

Supporting information.

Supporting information.

Supporting information.

## Data Availability

The data that support the findings of this study are available on request from the corresponding author. The data are not publicly available due to privacy or ethical restrictions.

## References

[jclp23769-bib-0001] Altimir, C. , M. Krause , G. de la Parra , et al. 2010. “Clients', Therapists', and Observers' Agreement on the Amount, Temporal Location, and Content of Psychotherapeutic Change and Its Relation to Outcome.” Psychotherapy Research: Journal of the Society for Psychotherapy Research 20, no. 4: 472–487. 10.1080/10503301003705871. 20552535

[jclp23769-bib-0002] APA . 2013. Diagnostic and Statistical Manual of Mental Disorders: DSM‐5^TM^ , *5th ed. (pp. xliv, 947)*. American Psychiatric Publishing, Inc. 10.1176/appi.books.9780890425596.

[jclp23769-bib-0003] Arcelus, J. , A. J. Mitchell , J. Wales , and S. Nielsen . 2011. “Mortality Rates in Patients With Anorexia Nervosa and Other Eating Disorders: A Meta‐Analysis of 36 Studies.” Archives of General Psychiatry 68, no. 7: 724–731. 10.1001/archgenpsychiatry.2011.74. 21727255

[jclp23769-bib-0004] Baier, A. L. , A. C. Kline , and N. C. Feeny . 2020. “Therapeutic Alliance as a Mediator of Change: A Systematic Review and Evaluation of Research.” Clinical Psychology Review 82: 101921. 10.1016/j.cpr.2020.101921. 33069096

[jclp23769-bib-0005] Bandini, S. , G. Antonelli , P. Moretti , S. Pampanelli , R. Quartesan , and G. Perriello . 2006. “Factors Affecting Dropout in Outpatient Eating Disorder Treatment.” Eating and Weight Disorders ‐ Studies on Anorexia, Bulimia and Obesity 11, no. 4: 179–184. 10.1007/BF03327569. 17272947

[jclp23769-bib-0006] Blythin, S. P. M. , H. L. Nicholson , V. G. Macintyre , J. M. Dickson , J. R. E. Fox , and P. J. Taylor . 2020. “Experiences of Shame and Guilt in Anorexia and Bulimia Nervosa: A Systematic Review.” Psychology and Psychotherapy: Theory, Research and Practice 93, no. 1: 134–159. 10.1111/papt.12198 30182527

[jclp23769-bib-0007] Bodell, L. P. , J. E. Wildes , Y. Cheng , et al. 2018. “Associations Between Race and Eating Disorder Symptom Trajectories in Black and White Girls.” Journal of Abnormal Child Psychology 46, no. 3: 625–638. 10.1007/s10802-017-0322-5. 28646354 PMC6063089

[jclp23769-bib-0008] Borelli, J. L. , C. Cohen , C. Pettit , et al. 2019. “Maternal and Child Sexual Abuse History: An Intergenerational Exploration of Children's Adjustment and Maternal Trauma‐Reflective Functioning.” Frontiers in Psychology 10. 10.3389/fpsyg.2019.01062. PMC6530340 31156503

[jclp23769-bib-0009] Braun, V. , and V. Clarke . 2006. “Using Thematic Analysis in Psychology.” Qualitative Research in Psychology 3, no. 2: 77–101.

[jclp23769-bib-0010] Braun, V. , V. Clarke , N. Hayfield , and G. Terry . 2019. “Thematic Analysis.” In Handbook of Research Methods in Health Social Sciences, edited by P. Liamputtong , 843–860. Springer Singapore. 10.1007/978-981-10-5251-4_103.

[jclp23769-bib-0011] Brown, A. , V. Mountford , and G. Waller . 2014. “Clinician and Practice Characteristics Influencing Delivery and Outcomes of the Early Part of Outpatient Cognitive Behavioural Therapy for Anorexia Nervosa.” The Cognitive Behaviour Therapist 7: e10. 10.1017/S1754470X14000105.

[jclp23769-bib-0012] Brown, A. , V. A. Mountford , and G. Waller . 2013. “Is the Therapeutic Alliance Overvalued in the Treatment of Eating Disorders?” International Journal of Eating Disorders 46, no. 8: 779–782. 10.1002/eat.22177. 23983066

[jclp23769-bib-0013] Bulik, C. M. , N. D. Berkman , K. A. Brownley , J. A. Sedway , and K. N. Lohr . 2007. “Anorexia Nervosa Treatment: A Systematic Review of Randomized Controlled Trials.” International Journal of Eating Disorders 40, no. 4: 310–320. 10.1002/eat.20367. 17370290

[jclp23769-bib-0014] Cain, C. L. , C. Taborda‐Whitt , M. Frazer , et al. 2017. “A Mixed Methods Study of Emotional Exhaustion: Energizing and Depleting Work Within an Innovative Healthcare Team.” Journal of Interprofessional Care 31, no. 6: 714–724. 28922038 10.1080/13561820.2017.1356809

[jclp23769-bib-0015] Clinton, D. N. 1996. “Why Do Eating Disorder Patients Drop Out?” Psychotherapy and Psychosomatics 65, no. 1: 29–35. 10.1159/000289028. 8838694

[jclp23769-bib-0016] Cook, S. C. , A. C. Schwartz , and N. J. Kaslow . 2017. “Evidence‐Based Psychotherapy: Advantages and Challenges.” Neurotherapeutics 14, no. 3: 537–545. 10.1007/s13311-017-0549-4. 28653278 PMC5509639

[jclp23769-bib-0017] Craig, P. , P. Dieppe , S. Macintyre , S. Michie , I. Nazareth , and M. Petticrew . 2011. Developing and Evaluating Complex Interventions. *Medical Research Council, UK*.

[jclp23769-bib-0018] Crits‐Christoph, P. , K. Baranackie , J. Kurcias , et al. 1991. “Meta‐Analysis of Therapist Effects in Psychotherapy Outcome Studies.” Psychotherapy Research 1, no. 2: 81–91. 10.1080/10503309112331335511.

[jclp23769-bib-0019] Crow, S. J. , C. B. Peterson , S. A. Swanson , et al. 2009. “Increased Mortality in Bulimia Nervosa and Other Eating Disorders.” American Journal of Psychiatry 166, no. 12: 1342–1346. 10.1176/appi.ajp.2009.09020247. 19833789

[jclp23769-bib-0020] De Castella, K. , P. Goldin , H. Jazaieri , M. Ziv , C. S. Dweck , and J. J. Gross . 2013. “Beliefs About Emotion: Links to Emotion Regulation, Well‐Being, and Psychological Distress.” Basic and Applied Social Psychology 35, no. 6: 497–505. 10.1080/01973533.2013.840632.

[jclp23769-bib-0021] De Castella, K. , M. J. Platow , M. Tamir , and J. J. Gross . 2018. “Beliefs About Emotion: Implications for Avoidance‐Based Emotion Regulation and Psychological Health.” Cognition and Emotion 32, no. 4: 773–795. 10.1080/02699931.2017.1353485. 28737108

[jclp23769-bib-0022] Dolhanty, J. , and L. S. Greenberg . 2009. “Emotion‐Focused Therapy in a Case of Anorexia Nervosa.” Clinical Psychology & Psychotherapy 16, no. 4: 366–382. 10.1002/cpp.624. 19639649

[jclp23769-bib-0023] Drinkwater, D. , S. Holttum , T. Lavender , H. Startup , and A. Oldershaw . 2022. “Seeing Through the Façade of Anorexia: A Grounded Theory of Emotional Change Processes Associated With Recovery From Anorexia Nervosa.” Frontiers in Psychiatry 13. 10.3389/fpsyt.2022.868586. PMC9263079 35815041

[jclp23769-bib-0024] Elliott, R. 2010. “Psychotherapy Change Process Research: Realizing the Promise.” Psychotherapy Research 20, no. 2: 123–135. 10.1080/10503300903470743. 20099202

[jclp23769-bib-0025] Elliott, R. 2011. “Qualitative Methods for Studying Psychotherapy Change Processes.” In Qualitative Research Methods in Mental Health and Psychotherapy, 69–81. John Wiley & Sons, Ltd. 10.1002/9781119973249.ch6.

[jclp23769-bib-0026] Elliott, R. , and L. S. Greenberg . 2007. “The Essence of Process‐Experiential/Emotion‐Focused Therapy.” American Journal of Psychotherapy 61, no. 3: 241–254. 10.1176/appi.psychotherapy.2007.61.3.241. 17985528

[jclp23769-bib-0027] Elliott, R. , and B. Rodgers . 2008. Client Change Interview Schedule (v5). Glasgow: University of Strathclyde.

[jclp23769-bib-0028] Flanagan, C. , T. Atkinson , and J. Young . 2020. “An Introduction to Schema Therapy: Origins, Overview, Research Status and Future Directions.” In Creative Methods in Schema Therapy. Routledge.

[jclp23769-bib-0029] Gordon, K. H. , M. M. Brattole , L. R. Wingate , and T. E. Joiner . 2006. “The Impact of Client Race on Clinician Detection of Eating Disorders.” Behavior Therapy 37, no. 4: 319–325. 10.1016/j.beth.2005.12.002. 17071210

[jclp23769-bib-0030] Graham, M. R. , S. Tierney , A. Chisholm , and J. R. E. Fox . 2020. “The Lived Experience of Working With People With Eating Disorders: A Meta‐Ethnography.” International Journal of Eating Disorders 53, no. 3: 422–441. 31904870 10.1002/eat.23215

[jclp23769-bib-0031] Greenberg, L. S. 1986. “Change Process Research.” Journal of Consulting and Clinical Psychology 54, no. 1: 4–9. 10.1037/0022-006X.54.1.4. 3958300

[jclp23769-bib-0032] Greenberg, L. S. 1991. “Research on the Process of Change.” Psychotherapy Research 1, no. 1: 3–16. 10.1080/10503309112331334011.

[jclp23769-bib-0033] Griffin, C. , P. Fenner , K. B. Landorf , and M. Cotchett . 2023. “Art Therapy and Eating Disorders: A Mixed Methods Feasibility Study.” The Arts in Psychotherapy 82: 101994. 10.1016/j.aip.2023.101994.

[jclp23769-bib-0034] Gülüm, İ. V. , and G. Soygüt . 2022. “Limited Reparenting as a Corrective Emotional Experience in Schema Therapy: A Preliminary Task Analysis.” Psychotherapy Research 32, no. 2: 263–276. 10.1080/10503307.2021.1921301. 33910484

[jclp23769-bib-0035] Hay, P. 2013. “A Systematic Review of Evidence for Psychological Treatments in Eating Disorders: 2005–2012.” International Journal of Eating Disorders 46, no. 5: 462–469. 10.1002/eat.22103. 23658093

[jclp23769-bib-0036] Ivanova, I. , and J. Watson . 2014. “Emotion‐Focused Therapy for Eating Disorders: Enhancing Emotional Processing.” Person‐Centered & Experiential Psychotherapies 13, no. 4: 278–293. 10.1080/14779757.2014.910132.

[jclp23769-bib-0037] Kazdin, A. E. , E. E. Fitzsimmons‐Craft , and D. E. Wilfley . 2017. “Addressing Critical Gaps in the Treatment of Eating Disorders.” International Journal of Eating Disorders 50, no. 3: 170–189. 10.1002/eat.22670. 28102908 PMC6169314

[jclp23769-bib-0038] Keeler, J. L. , R. Chami , V. Cardi , et al. 2022. “App‐Based Food‐Specific Inhibitory Control Training as an Adjunct to Treatment as Usual in Binge‐Type Eating Disorders: A Feasibility Trial.” Appetite 168: 105788. 10.1016/j.appet.2021.105788. 34728250

[jclp23769-bib-0039] Keltner, D. , and J. J. Gross . 1999. “Functional Accounts of Emotions.” Cognition & Emotion 13, no. 5: 467–480.

[jclp23769-bib-0040] Kendler, K. S. , and J. Campbell . 2009. “Interventionist Causal Models in Psychiatry: Repositioning the Mind–Body Problem.” Psychological Medicine 39, no. 6: 881–887. 18845010 10.1017/S0033291708004467

[jclp23769-bib-0041] Leppanen, J. , D. Brown , H. McLinden , S. Williams , and K. Tchanturia . 2022. “The Role of Emotion Regulation in Eating Disorders: A Network Meta‐Analysis Approach.” Frontiers in Psychiatry 13. 10.3389/fpsyt.2022.793094. PMC8904925 35280172

[jclp23769-bib-0042] Malik‐Smith, S. , M. Callanan , A. Pascual‐Leone , C. Papastavrou Brooks , and A. Oldershaw . In prep. Exploring How the Process of Emotional Change is Associated With Recovery During an Adapted Emotion‐Focused Therapy for Adults With Anorexia Nervosa.

[jclp23769-bib-0043] McDonald, S. , A. J. Williams , P. Barr , N. McNamara , and M. Marriott . 2021. “Service User and Eating Disorder Therapist Views on Anorexia Nervosa Recovery Criteria.” Psychology and Psychotherapy: Theory, Research and Practice 94, no. 3: 721–736. 10.1111/papt.12340. PMC8451855 33761183

[jclp23769-bib-0044] Morlino, M. , G. Di Pietro , R. Tuccillo , et al. 2007. “Drop‐Out Rate in Eating Disorders: Could It Be a Function of Patient‐Therapist Relationship?” Eating and Weight Disorders ‐ Studies on Anorexia, Bulimia and Obesity 12, no. 3: e64–e67. 10.1007/BF03327645. 17984632

[jclp23769-bib-0045] Morse, G. , M. P. Salyers , A. L. Rollins , M. Monroe‐DeVita , and C. Pfahler . 2012. “Burnout in Mental Health Services: A Review of the Problem and Its Remediation.” Administration and Policy in Mental Health and Mental Health Services Research 39, no. 5: 341–352. 10.1007/s10488-011-0352-1. 21533847 PMC3156844

[jclp23769-bib-0046] Murray, S. B. , E. Pila , S. Griffiths , and D. Le Grange . 2017. “When Illness Severity and Research Dollars Do Not Align: Are We Overlooking Eating Disorders?” World Psychiatry : Official Journal of the World Psychiatric Association (WPA) 16, no. 3: 321. 10.1002/wps.20465. 28941116 PMC5608830

[jclp23769-bib-0047] NICE . 2017. Anorexia Nervosa: Treatment for Adults. Information for the Public. Eating disorders: Recognition and Treatment. Guidance. NICE. https://www.nice.org.uk/guidance/ng69/ifp/chapter/Anorexia-nervosa-treatment-for-adults.

[jclp23769-bib-0048] Nødtvedt, Ø. O. , P.‐E. Binder , S. H. Stige , E. Schanche , J. R. Stiegler , and A. Hjeltnes . 2019. “‘You Feel They Have a Heart and Are Not Afraid to Show It’: Exploring How Clients Experience the Therapeutic Relationship in Emotion‐Focused Therapy.” Frontiers in Psychology 10. 10.3389/fpsyg.2019.01996. PMC6737069 31572255

[jclp23769-bib-0049] Oldershaw, A. , R. S. Basra , T. Lavender , and H. Startup . 2024. “Specialist Psychotherapy With Emotion for Anorexia in Kent and Sussex: An Intervention Development and Non‐Randomised Single Arm Feasibility Trial.” European Eating Disorders Review 32, no. 2: 215–229. 10.1002/erv.3034. 37815048

[jclp23769-bib-0050] Oldershaw, A. , H. DeJong , D. Hambrook , et al. 2012. “Emotional Processing Following Recovery From Anorexia Nervosa.” European Eating Disorders Review 20, no. 6: 502–509. 10.1002/erv.2153. 22241653

[jclp23769-bib-0051] Oldershaw, A. , T. Lavender , R. Basra , and H. Startup . 2022. “SPEAKS Study: Study Protocol of a Multisite Feasibility Trial of the Specialist Psychotherapy With Emotion for Anorexia in Kent and Sussex (SPEAKS) Intervention for Outpatients With Anorexia Nervosa or Otherwise Specified Feeding and Eating Disorders, Anorexia Nervosa Type.” BMJ Open 12, no. 2: e050350. 10.1136/bmjopen-2021-050350 PMC8867350 35193902

[jclp23769-bib-0052] Oldershaw, A. , T. Lavender , H. Sallis , D. Stahl , and U. Schmidt . 2015. “Emotion Generation and Regulation in Anorexia Nervosa: A Systematic Review and Meta‐Analysis of Self‐Report Data.” Clinical Psychology Review 39: 83–95. 26043394 10.1016/j.cpr.2015.04.005

[jclp23769-bib-0053] Oldershaw, A. , and H. Startup . 2020. “Building the Healthy Adult in Eating Disorders: A Schema Mode and Emotion‐Focused Therapy Approach for Anorexia Nervosa.” In Creative Methods in Schema Therapy. Routledge.

[jclp23769-bib-0054] Oldershaw, A. , and H. Startup . 2023. “Emotion Focused Schema Therapy for Anorexia Nervosa: The SPEAKS Approach.” In Eating Disorders: An International Comprehensive View, edited by P. Robinson , T. Wade , B. Herpertz‐Dahlmann , F. Fernandez‐Aranda , J. Treasure , and S. Wonderlich . Springer International Publishing. 10.1007/978-3-030-97416-9_12-1.

[jclp23769-bib-0055] Pascual‐Leone, A. 2018. “How Clients ‘Change Emotion With Emotion’: A Programme of Research on Emotional Processing.” Psychotherapy Research 28, no. 2: 165–182. 28714778 10.1080/10503307.2017.1349350

[jclp23769-bib-0056] Pos, A. E. , and L. S. Greenberg . 2007. “Emotion‐Focused Therapy: The Transforming Power of Affect.” Journal of Contemporary Psychotherapy 37, no. 1: 25–31. 10.1007/s10879-006-9031-z.

[jclp23769-bib-0057] Puttevils, L. , M.‐A. Vanderhasselt , P. Horczak , and M. Vervaet . 2021. “Differences in the Use of Emotion Regulation Strategies Between Anorexia and Bulimia Nervosa: A Systematic Review and Meta‐Analysis.” Comprehensive Psychiatry 109: 152262. 10.1016/j.comppsych.2021.152262. 34265598

[jclp23769-bib-0058] Rennick, A. , C. Papastavrou Brooks , R. Singh Basra , H. Startup , T. Lavender , and A. Oldershaw . 2024. “Acceptability of Specialist Psychotherapy With Emotion for Anorexia in Kent and Sussex (SPEAKS): A Novel Intervention for Anorexia Nervosa.” International Journal of Eating Disorders 57: 611–623. 10.1002/eat.24139. 38258350

[jclp23769-bib-0059] Robinson, A. L. , J. Dolhanty , and L. Greenberg . 2015. “Emotion‐Focused Family Therapy for Eating Disorders in Children and Adolescents.” Clinical Psychology & Psychotherapy 22, no. 1: 75–82. 10.1002/cpp.1861. 23913713

[jclp23769-bib-0060] Sala, M. , A. Heard , and E. A. Black . 2016. “Emotion‐Focused Treatments for Anorexia Nervosa: A Systematic Review of the Literature.” Eating and Weight Disorders ‐ Studies on Anorexia, Bulimia and Obesity 21, no. 2: 147–164. 10.1007/s40519-016-0257-9. 26886827

[jclp23769-bib-0061] Sekhon, M. , M. Cartwright , and J. J. Francis . 2017. “Acceptability of Healthcare Interventions: An Overview of Reviews and Development of a Theoretical Framework.” BMC Health Services Research 17, no. 1: 88. 10.1186/s12913-017-2031-8. 28126032 PMC5267473

[jclp23769-bib-0062] Skivington, K. , L. Matthews , S. A. Simpson , et al. 2021. “A New Framework for Developing and Evaluating Complex Interventions: Update of Medical Research Council Guidance.” BMJ (London) 374. 10.1136/bmj.n2061. PMC8482308 34593508

[jclp23769-bib-0063] Solmi, M. , T. D. Wade , S. Byrne , et al. 2021. “Comparative Efficacy and Acceptability of Psychological Interventions for the Treatment of Adult Outpatients With Anorexia Nervosa: A Systematic Review and Network Meta‐Analysis.” The Lancet Psychiatry 8, no. 3: 215–224. 10.1016/S2215-0366(20)30566-6. 33600749

[jclp23769-bib-0064] Stiegler, J. R. , H. Molde , and E. Schanche . 2018. “Does an Emotion‐Focused Two‐Chair Dialogue Add to the Therapeutic Effect of the Empathic Attunement to Affect?” Clinical Psychology & Psychotherapy 25, no. 1: 86. 10.1002/cpp.2144. 28960601

[jclp23769-bib-0065] Thapliyal, P. , D. Mitchison , and P. Hay . 2017. “Insights Into the Experiences of Treatment for An Eating Disorder in Men: A Qualitative Study of Autobiographies.” Behavioral Sciences 7, no. 2: 38. 10.3390/bs7020038. 28621727 PMC5485468

[jclp23769-bib-0066] van Hoeken, D. , and H. W. Hoek . 2020. “Review of the Burden of Eating Disorders: Mortality, Disability, Costs, Quality of Life, and Family Burden.” Current Opinion in Psychiatry 33, no. 6: 521–527. 10.1097/YCO.0000000000000641. 32796186 PMC7575017

[jclp23769-bib-0067] Varpio, L. , E. Paradis , S. Uijtdehaage , and M. Young . 2020. “The Distinctions Between Theory, Theoretical Framework, and Conceptual Framework.” Academic Medicine 95, no. 7: 989–994. 31725464 10.1097/ACM.0000000000003075

[jclp23769-bib-0068] Watson, J. C. 2018. “Empathy and Responsiveness in Emotion‐Focused Therapy.” In Developing the Therapeutic Relationship: Integrating Case Studies, Research, and Practice, 235–255. American Psychological Association. 10.1037/0000093-011.

[jclp23769-bib-0069] Wayda‐Zalewska, M. , P. Grzegorzewski , E. Kot , E. Skimina , P. S. Santangelo , and K. Kucharska . 2022. “Emotion Dynamics and Emotion Regulation in Anorexia Nervosa: A Systematic Review of Ecological Momentary Assessment Studies.” International Journal of Environmental Research and Public Health 19, no. 20: 13659. 10.3390/ijerph192013659. 36294238 PMC9603728

[jclp23769-bib-0070] Williams, K. , J. King , and J. R. E. Fox . 2016. “Sense of Self and Anorexia Nervosa: A Grounded Theory.” Psychology and Psychotherapy: Theory, Research and Practice 89, no. 2: 211–228. 10.1111/papt.12068. 26179295

[jclp23769-bib-0071] Wollburg, E. , B. Meyer , B. Osen , and B. Löwe . 2013. “Psychological Change Mechanisms in Anorexia Nervosa Treatments: How Much Do We Know?” Journal of Clinical Psychology 69, no. 7: 762–773. 23349069 10.1002/jclp.21945

[jclp23769-bib-0072] Wong, V. , E. M. Koithan , B. Santos , A. Johnson , and A. Haynos . 2023. Does Evidence Support the Emotion Regulation Model of Anorexia Nervosa? A Systematic Review of Over a Decade of Research. 10.31234/osf.io/6s4t9. PMC12716886 41426278

[jclp23769-bib-0073] Wooldridge, T. , and P. Lytle . 2012. “An Overview of Anorexia Nervosa in Males.” Eating Disorders 20, no. 5: 368–378. 10.1080/10640266.2012.715515. 22985234

[jclp23769-bib-0074] Zaitsoff, S. , R. Pullmer , M. Cyr , and H. Aime . 2015. “The Role of the Therapeutic Alliance in Eating Disorder Treatment Outcomes: A Systematic Review.” Eating Disorders 23, no. 2: 99–114. 10.1080/10640266.2014.964623. 25330409

