# Supplementary material for: “It's OK for Me to Cry”: Client and Therapist Perspectives on Change Processes in SPEAKS Therapy for Anorexia Nervosa
Source: J Clin Psychol. 2025 Jan 13;81(5):298–310. doi: 10.1002/jclp.23769 (PMC11971651; doi:10.1002/jclp.23769)
Supplement: Supplementary file 1 — Supporting information. [file JCLP-81-298-s003.docx]

| **Qualitative Interview Schedule for therapists** 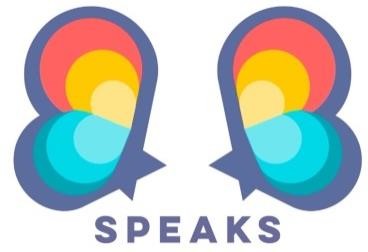 Version 1.0 (11/2020)  With reference to the Client Change Interview Protocol by R. Elliott (2011)  Follow up questions given in green. | |
| --- | --- |
| **Topic** | **Question(s)** |
| **Situating questions** | (1a) How are you doing now in general?  (1b) What has delivering the SPEAKS therapy been like for you so far? How has it felt to deliver this therapy? |
| **Questions about the therapy** | |
| **Perceived change** | (2a, b, c) Can you tell me about any ways in which things have changed for your patients since starting SPEAKS?  Are there any changes that you felt didn’t happen that you would have liked to see? Are there any changes that you felt didn’t happen that  you feel your clients would have liked to see? |
| **Valued psychotherapeutic targets and techniques** | (4) What do you think caused these changes for patients, either in and out of therapy?  (7) (If you feel SPEAKS has helped them) what do you think have been the most helpful parts of the therapy?  As you know SPEAKS uses some techniques, such as like thinking about different parts of the patient or using chairs.  What did you think of the techniques used in SPEAKS? Are there any things in particular that were especially valuable, or that you’ll take away from therapy? Can you give examples? |
| **Perceived impact on emotion** | What impact do you think SPEAKS has had on how your patients perceive or think about emotions?  Can you give me an example?  Has this been useful? In what ways?  What impact do you think it has it had on how they manage their feelings?  Can you give me an example?  Has this been useful? In what ways? |
| **Any unhelpful or unnecessary elements** | What has been hindering or unhelpful or negative or disappointing about the SPEAKS intervention?  Were there things that were difficult for patients which they managed to overcome?  Was there anything missing from the therapy? |
| **Impact on therapists** | Can you describe your experience of being a SPEAKS therapist? How did you feel about being a SPEAKS therapist during therapy? How do you feel about having been a SPEAKS therapist now?  In what ways did delivering the SPEAKS intervention differ from applying other psychological models? Was there anything that was particularly helpful or unhelpful? What did you learn from this? |
| **Effectiveness of supervision in identifying and managing personal factors** | How was the experience of supervision whilst using the SPEAKS model?  Was there anything in particular that was helpful about supervision? Was there anything hindering or unhelpful or negative or disappointing about SPEAKS supervision?  Were there things that were difficult but were actually ok or helped? Was there anything missing from the supervision? |

| **Intervention implementation** | What did you think of the way in which the SPEAKS therapy was delivered?  What did you think of delivering SPEAKS as an online therapy? Why? Have you had any concerns? |
| --- | --- |
| **Questions about the research trial** | |
| **Acceptability of a future RCT of SPEAKS (design, such as willingness to be randomised, selected measures and the use of SSCM as a standard comparator)** | (10) What has it been like to deliver therapy as part of a research study?  What has been helpful or hindering about this in terms of your therapeutic practice or the intervention (as compared with delivering therapy outside of a research study)?  We are thinking of running a larger trial of SPEAKS in the future. What do you think about this?  In this case people would be randomly assigned to receive SPEAKS or something else. What do you think about people being randomly assigned to therapy? Do you think people with anorexia would be willing to take part? Could you explain why?  Which variables of change do you think are most important to capture for this client group and/or from SPEAKS therapy? |
| **Including ease of integration into services** | Do you think being involved in the SPEAKS study affected the care that your clients received from (Kent/Sussex) Eating Disorder service? In what way? Were their needs by the service as a whole?  Were your needs as a trial therapist met during your involvement with the trial?  What systems or arrangements within the study or the wider service were helpful, unhelpful or missing in terms of providing the support you needed as a SPEAKS trial therapist?  Is there anything else that did or didn’t happen during your time in the study that you feel it is useful to reflect on or for us to know about?  Is there anything about the SPEAKS study that you think we should know about for future research? |
