# Supplementary material for: “It's OK for Me to Cry”: Client and Therapist Perspectives on Change Processes in SPEAKS Therapy for Anorexia Nervosa
Source: J Clin Psychol. 2025 Jan 13;81(5):298–310. doi: 10.1002/jclp.23769 (PMC11971651; doi:10.1002/jclp.23769)
Supplement: Supplementary file 2 — Supporting information. [file JCLP-81-298-s001.docx]

| **Qualitative Interview Schedule for participants** 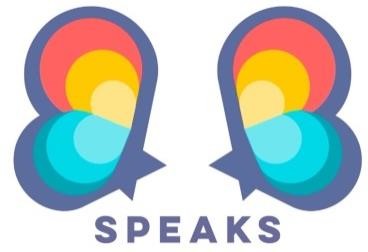 Version 1.0 (11/2020)  With reference to the Client Change Interview Protocol by R. Elliott (2011)  Follow up questions given in burgundy/red. | |
| --- | --- |
| **Topic** | **Question(s)** |
| **Situating questions** | (1a) How are you doing now in general?  (1b) What has therapy been like for you so far? How has it felt to be in therapy? |
| **Questions about the therapy** | |
| **Perceived change** | (2a, b, c) Can you tell me about any ways in which things have changed for you since starting SPEAKS?  Are there things that haven’t changed that you’d like to change still? |
| **Valued psychotherapeutic targets and techniques** | (4) What has caused these changes for you, either in and out of therapy  (7) (If you feel SPEAKS has helped you) what do you think have been the most helpful parts of the therapy?  SPEAKS uses some techniques that might have been new to you, like thinking about different parts of you or using chairs.  What did you think of the techniques used in SPEAKS? Are there any things in particular that were especially valuable, or that stay with you and you’ll take away from therapy? Can you give examples? |
| **Perceived impact on emotion** | What impact has SPEAKS had on how you perceive or think about your emotions?  Can you give me an example?  Has this been useful? In what ways?  What impact has it had on how you manage your feelings? Can you give me an example?  Has this been useful? In what ways? |
| **Any unhelpful or unnecessary elements** | What has been hindering or unhelpful or negative or disappointing about SPEAKS?  Were there things that were difficult but were actually ok or helped? Was there anything missing from the therapy? |
| **Participant experiences of therapists** | Can you describe your experience of your SPEAKS therapist? How did you feel about your SPEAKS therapist during therapy? How do you feel about your SPEAKS therapist now?  Was there anything about the way your therapist related or interacted with you that was helpful or unhelpful? |
| **Intervention implementation** | What did you think of the way in which the SPEAKS therapy was delivered?  What did you think of having SPEAKS as an online therapy? Why? Have you had any concerns? |

| **Questions about the research trial** | |
| --- | --- |
| **Acceptability of a future RCT of SPEAKS (design, such as willingness to be randomised, selected measures and the use of SSCM as a standard comparator)** | (10) What has it been like to be involved in a research study?  What has been helpful or hindering about this?  We are thinking of running a larger trial of SPEAKS in the future. What do you think about this?  In this case people would be randomly assigned to receive SPEAKS or something else. What do you think about people being randomly assigned to therapy? Do you think other people in your position would be willing to take part? Could you explain why?  Do you have any feedback on the questionnaires we used in this study? Were there any that particularly connected with your experience?  Is there anything else we did or didn’t do during your time in the  study? Is there anything about the SPEAKS study that you think we should know about for future research? |
| **Including ease of integration into services** | Do you think being involved in the SPEAKS study affected the care you received from (Kent/Sussex) Eating Disorder service? In what way? Were your needs by the service as a whole? |
