# Supplementary material for: “It's OK for Me to Cry”: Client and Therapist Perspectives on Change Processes in SPEAKS Therapy for Anorexia Nervosa
Source: J Clin Psychol. 2025 Jan 13;81(5):298–310. doi: 10.1002/jclp.23769 (PMC11971651; doi:10.1002/jclp.23769)
Supplement: Supplementary file 3 — Supporting information. [file JCLP-81-298-s002.docx]

**Appendix 3: Coding Tree for SPEAKS Qualitative Analysis**

Acceptability of SPEAKS Components and Processes

- Chairwork
- Use of toys/formulation
- Safe bubble
- Ending letters
- Frequency and duration of therapy
- Sessions being recorded
- Use of questionnaires

Impact on the individual

- Emotions
  - Normalising negative feelings
  - Identifying and managing emotions
  - Understanding other’s emotions
  - Emotionally difficult process
- Self
  - Understanding triggers of ED
  - Less self-doubt, more confidence
  - Allowing time to focus on self
  - Worries about end of therapy
- Physical health
  - Impact on weight
  - Impact on relationship with food/body
  - Lack of focus on food in therapy

Therapeutic relationship

- Trust and safe space to open up
- Respect for qualifications
- Positive relationship highly valued
- Not feeling heard by therapist/feeling patronised

Delivery method of SPEAKS

- Convenience of being online
- No difference online compared to F2F
- Confidentiality worries over online delivery
- Not having space to decompress at home
- Wanting connection with other therapists

Therapist-specific factors

- Confidence with delivering techniques
- Need for supervision (individual and group) & training
- Concern around resources
  - Practical resources
  - Emotional resources
- Learning curve’ - different to models they are used to

Experience of being involved in a research trial

- Clients
  - Feeling special/ pioneering
  - Wanting to help others
  - Access to things that you wouldn’t normally have had
  - Lack of choice
  - Impact on usual care
  - Completing questionnaires
- Therapists
  - Extra pressure/nerve-wracking
  - Recording sessions
  - Didn’t feel like a research trial
  - Receiving feedback

Future Trial

- Key variables for a future trial
- Willingness to be randomized
- Scaling the study
